# A Comprehensive Study on the Dynamic Change of Thermal Behavior During Lignocellulose Pyrolysis Catalyzed by Plant-Rich Metallic Elements

**DOI:** 10.3389/fpls.2021.665470

**Published:** 2021-11-05

**Authors:** Jiawei Huang, Biao Zheng, Zhou Hong, Peiyao Ouyang, Yuanhua Li, Aimin Wu, Huiling Li

**Affiliations:** ^1^State Key Laboratory for Conservation and Utilization of Subtropical Agro-bioresources, South China Agricultural University, Guangzhou, China; ^2^Guangdong Key Laboratory for Innovative Development and Utilization of Forest Plant Germplasm, College of Forestry and Landscape Architecture, South China Agricultural University, Guangzhou, China; ^3^Research Institute of Tropical Forestry, Chinese Academy of Forestry, Guangzhou, China; ^4^School of Chemistry and Materials Engineering, Huizhou University, Huizhou, China

**Keywords:** pyrolysis, plant-rich metallic element, thermal behavior, eucalyptus, bamboo

## Abstract

Evaluating the pyrolysis of lignocellulose *via* theoretical and computational approaches is of great importance for the efficient utilization of biomass. In this work, the dynamic changes in physicochemical properties of eucalyptus and bamboo during plant-rich metallic element-catalyzed pyrolysis process were investigated, and their thermal decomposition behaviors were explored by kinetic analysis. Results showed that the metal absorption capacity and thermal stability of eucalyptus were better than those of bamboo. The temperatures corresponding to the initial devolatilization and the highest weight loss value of eucalyptus/bamboo decreased in the catalysis order of Mg > Fe > Ca > Cu > K > Na. Fourier-transform infrared (FT-IR) results showed that the thermal stability of ester bond of glucuronoarabinoxylan was higher than that of acetyl groups. The maximum weight loss rate could be observed for samples with the lowest metal-loaded concentration (5%). Moreover, Mg and Fe presented the better catalytic performance for facilitating the lignocellulose pyrolysis in comparison with other investigated metallic elements.

## Introduction

With the draining of fossil fuels and the increasing demand of energy, extensive studies had been conducted on the production of fuels and chemicals from alternatives to displace petroleum-derived products. Lignocellulose, which is abundant and cheap, can be served as an important renewable feedstock in the predominant carbon-neutral sources of biofuels and biomaterials (Huang et al., 2019; Soltanian et al., 2020). Plants possess metal-rich properties that usually absorb over much metallic elements for growth (Kroukamp et al., 2016; Rezania et al., 2016; Batool and Saleh, 2020). Therefore, the over-observed metallic elements should be used fully to enhance the utilization efficiency of lignocellulose.

Pyrolysis is a fundamental principle of various thermochemical conversion processes of lignocellulose, and it is one of the key pathways for the efficient generation of fuels and chemicals from abundant materials (George et al., 2006; Cao et al., 2014). However, the pyrolysis process of lignocellulose is extremely complex. It can be influenced by many factors and generally goes through a series of reactions. Lignocellulose is primarily comprised of cellulose, hemicellulose, and lignin with small traces of other components (Wang et al., 2020). Incumbent approaches for biomass pyrolysis involve mainly four stages: moisture evaporation (100°C), hemicellulose decomposition (220–315°C), cellulose decomposition (314–400°C), and lignin decomposition (160–900°C) (Yang et al., 2007; Chen et al., 2019). The pyrolysis behavior of lignocellulose varied in accordance with many factors, such as the plant species, catalyst types, etc. Thus, monitoring changes during lignocellulose pyrolysis is vitally important to efficiently control and regulate the process (Chen et al., 2014; Gao et al., 2015; Naron et al., 2019; Zhang et al., 2019).

Small traces of other components of lignocellulose include metallic elements that exist in the form of oxides or salts, such as potassium (K), calcium (Ca), and magnesium (Mg), varying from the growth environment and plant species. Previous studies demonstrated that alkali metals in lignocellulose exerted a catalytic effect during the pyrolysis of plant biomass (Shi et al., 2012; Jiang et al., 2013; Oudenhoven et al., 2015). Gao et al. (2015) indicated that both inherent and external metallic elements had influences on the products formation during straw pyrolysis. Studies showed that the impregnation of rice straw with CaCl_2_ and MgCl_2_ solution not only greatly promoted the pyrolysis of lignocellulose but also reduced the reaction temperature for desired products. Saddawi et al. (2012) demonstrated that the apparent activation energy for willow pyrolysis was reduced in the presence of alkali metals (K, Na) (Saddawi et al., 2012). Although the catalytic pyrolysis of lignocellulose *via* metal oxides/salts has been investigated by various studies previously in the aspect of reaction conditions and apparent activation energy, detailed information on the comprehensive study of the effect of metallic elements that are enriched in plant growth on the dynamic changes of thermal behavior during different lignocellulose pyrolysis is inadequate.

Eucalyptus is one of the fast-growing and high-density tree species in the world with a fixed biomass efficiently (Sgroi et al., 2015). Bamboo, which is regarded as an alternative to wood in many aspects, is also a fast-growing perennial herbaceous plant with a large phytomass (Oyedun et al., 2013). Both eucalyptus and bamboo are economic species, which are usually used for industrial purpose. In the present study, a comprehensive understanding to the effects of plant-rich metallic elements on the changes in physicochemical properties of eucalyptus and bamboo during pyrolysis was prospected. The metallic elements used in this study were selected according to the plant growth requirements as well as metallic elements those may be enriched in the metal-contaminated soil. Kinetic models of the catalytic pyrolysis of eucalyptus and bamboo after plant-rich metal-loaded were established. This work provided a feasible theoretical basis for the efficient utilization of plants intrinsically enriched metals to catalyze themselves pyrolysis.

## Materials and Methods

### Materials

Eucalyptus trunk was obtained from Chinese Academy of Forestry (Guangzhou, China). Bamboo stems were obtained from Zhejiang A&F University (Hangzhou, China). Before experiments, the samples were grounded to particle size >80 mesh and dried at 65°C for 4 days. Wax and other extracts of eucalyptus and bamboo were removed by refluxing with acetone/ethanol (2:1, v/v) at 65°C for 8 h in a Soxhlet apparatus, and the samples were washed with DI water and oven-dried at 60°C to constant weight. Chemical compositions of eucalyptus and bamboo were measured by following the established National Renewable Energy Laboratory standard analytic procedure (NREL/TP-510-42618) (Sluiter et al., 2008). The contents of ash, volatile, and fixed carbon in raw materials were determined according to the existing literature (Cai et al., 2017). Acid-washed samples were prepared by the acid wash method. In brief, lignocellulosic particles were impregnated by 14.44% hydrochloric acid for 12 h, then washed by DI water until pH of the eluent became neutral. The obtained acid-washed samples were oven-dried at 60°C to constant weight. Extracted materials without acid-washed were named as REP (eucalyptus) and RBB (bamboo), while acid-washed samples were named as EP (acid-washed eucalyptus) and BB (acid-washed bamboo). Metal-loaded biomass was prepared by the wet impregnation method. Acid-washed materials were immersed by NaCl, KCl, CaCl_2_, MgCl_2_, CuCl_2_, and FeCl_3_ aqueous solutions for 24 h with the metal weight percentage of 5, 10, and 15 wt%, respectively. Solid fraction was washed by DI water and oven-dried at 65°C for 6 h. Metal-loaded material was annotated as A-B-C, where A was the plant species (EP or BB), B was the metallic element (Na, K, Ca, Mg, Cu, Fe), and C was the metal weight percentage (wt%).

All chemicals, which were of AR grade, were purchased from Shanghai Sigma-Aldrich Trading Co. Ltd and used without any purification (Shanghai, China). Deionized water was used to prepare all solutions. All of the chemicals and reagents were used without any purification.

### Analytical Methods

Water retention value (WRV) of EP and BB was measured according to the literature (Sherpa et al., 2018). In brief, 1 g of sample was mixed with 5 mL of DI water and shaken at 150 rpm for 1 h at room temperature. The mixture was filtered subsequently. The solid fraction was transferred into a small non-woven bag and soaked in DI water for 2 h at room temperature, followed by centrifugation at 1,830 rpm for 15 min. Solid products were oven-dried at 105°C for 24 h. WRV was defined as the following equation:


(1) $$\text{WRV}(\frac{\text{g}}{\text{g}}\text{dry biomass})\text{=}\frac{{\text{WRV}}_{\text{wet }}{-\text{ WRV}}_{\text{dry}}}{{\text{WRV}}_{\text{dry}}}$$

where W_wet_ is the weight of solid products after centrifugation; W_dry_ is the weight of the final oven-dried samples.

Atomic absorption spectrometer (AAS, 220FS, Varian, Australia) was used to analyze the contents of metallic elements in treated and untreated samples. About 300 mg of sample was dissolved in the mixture containing 7 mL of nitric acid and 2 mL of hydrogen peroxide. Standard curve was used to determine the metallic element contents in the samples. Metal element removal rate of acid-washed was calculated. The removal rate was defined as the following equation:


(2) $$\text{Removal rate = }\frac{{\text{C}}_{\text{R}}-\text{C}}{{\text{C}}_{\text{R}}}\times \text{100}\%$$

where CR is the metal element content of raw materials without acid wash, and C is the metal element content of raw materials with acid wash.

The pyrolysis behavior of the raw materials, acid-washed materials, and metal-loaded samples was performed by a thermogravimetric analyzer (TGA, Q500, TA, America). Sample (5–10 mg) was distributed evenly into a ceramic crucible and heated up to the desired temperature (700°C) with a constant heating rate of 20°C/min. Purified nitrogen (99.9%) was used as the carrier gas at a flow rate of 40 mL/min. In order to investigate the dynamic structure change during pyrolysis, pyrolyzed samples were prepared by the pyrolysis method in a tube furnace with different pyrolyzed temperature with 50 mg granular sample dosage. The heating rate and the nitrogen flow rate were the same as those of TG analysis. The final temperature was set as 160, 220, 280, 340, 400, 460, 520, 580, 640, and 700°C. The structural changes of eucalyptus and bamboo during pyrolysis were measured by Fourier-transform infrared (FT-IR) spectra (Bruker, Tensor II, Germany) using a KBr disk containing 1% ground sample. Fourteen scans were collected in the range of 500–2,500 cm^−1^ at a resolution of 4 cm^−1^.

### Kinetic Analysis

Biomass pyrolysis is a complex process that includes a series of physical and chemical changes. Previous studies showed that biomass pyrolysis is a non-independent process (Li and Suzuki, 2009; Sutcu and Piskin, 2009; Yorulmaz and Atimtay, 2009). Coast–Redfern integral method with the first-order reaction assumption was widely used to calculate apparent activation energy (Ea) of biomass pyrolysis (Naqvi et al., 2019). A non-isothermal kinetic equation of the biomass pyrolysis reaction was applied as Equation. (3):


(3) $$\frac{\text{d}\alpha }{\text{1}-\alpha }\text{=}\frac{\text{A}}{\beta }\exp(-\frac{\text{Ea}}{\text{RT}})\text{dT}$$

where A is the pre-exponential factor (s^−1^), Ea is the apparent activation energy (kJ/mol), β is the linear heating rate for non-isothermal conditions (20°C/min), R is the gas constant of 8.314 J/(mol·K), and T is the absolute temperature (K). Moreover, α refers to the conversion rate of raw material, which could be calculated by Equation. (4):


(4) $$\alpha \text{=}\frac{{\text{W}}_{\text{0}}-{\text{W}}_{\text{t}}}{{\text{W}}_{\text{0}}-{\text{W}}_{\text{f}}}$$

where W_0_ is the initial sample weight for TG analysis, W_t_ refers to the weight at corresponding time t, and W_f_ is the weight of pyrolysis residues.

Equation. (3) could be written as follows:


(5) $$\int_{0}^{\alpha }\frac{\text{d}\alpha }{1-\alpha }=\frac{A}{\beta }\int_{{\text{T}}_{0}}^{\text{T}}\exp(-\frac{\text{Ea}}{\text{RT}})\text{ dT}$$

(6) $$\begin{array}{r} \ln\left(1-\text{α}\right)={\text{T}}^{2}\left(\frac{AR}{\beta Ea}\times \frac{Ea-2RT}{Ea}\right)\times \exp\left(-\frac{Ea}{RT}\right) \end{array}$$

(7) $$\ln\left[-\frac{\ln(\text{1-}\alpha )}{{\text{T}}^{\text{2}}}\right]\text{=}-\frac{\text{Ea}}{\text{RT}}\text{+}\ln\frac{\text{AR}}{\beta \text{Ea}}$$

A linear equation of Y=M+NX was obtained by plotting of Equation. (8).

where,


(8) $$\text{Y = }\ln\left[-\frac{\ln(1-\alpha )}{{\text{T}}^{\text{2}}}\right],\text{ M = }\ln\frac{\text{AR}}{\beta \text{Ea}},\text{ N = }-\frac{\text{Ea}}{\text{R}},\text{X = }\frac{\text{1}}{\text{T}}\;$$

## Results and Discussions

### Physicochemical Properties of Raw Materials

The compositions of raw materials (REP and RBB) are shown in Table 1. The cellulose, hemicellulose, and lignin contents of REP were 45.62, 18.72, and 31.59%, respectively, while RBB consisted of 46.24% cellulose, 14.37% hemicellulose, and 36.34% lignin. WRV was a parameter to reflect the swelling ability of biomass. It could be used as an indicator of the accessibility of interior surface. The WRV of REP and RBB was 241.58 and 221.27%, respectively. Therefore, we conjectured that the adsorption capacity of REP may be better than that of RBB, which was also confirmed by the following metal absorption results. The volatile matter of REP was 79.34%, which was a little higher than that of RBB (76.79%). However, the opposite trend was observed for fixed carbon. The fixed carbon contents of RBB and REP were 19.10 and 15.30%, respectively. Difference in volatile matter and fixed carbon contents between REP and RBB may be ascribed to their different chemical compositions. According to the literature, the higher carbohydrates content resulted in the larger volatile percentage (Demirbas, 2003). The more lignin content in the lignocellulose is, the higher fixed carbon percentage could be obtained (Demirbas, 2003). The ash contents of REP and RBB were 0.22 and 1.37%, respectively. The diversity of ash content between REP and RBB may be due to their different growth environment and physiological characteristics (Fahmi et al., 2007).

**Table 1:** Composition analysis of raw materials.

**Sample**	**Lignin (%)**	**Cellulose (%)**	**Hemicellulose (%)**	**WRV (g/g)**	**Volatile matter (%)**	**Fixed carbon (%)**	**Ash (%)**
EP	31.59 ± 1.32	45.62 ± 0.95	18.72 ± 0.88	241.58 ± 4.21	79.34 ± 2.12	15.30 ± 3.20	0.22 ± 0.012
BB	36.34 ± 1.54	46.24 ± 0.78	14.37 ± 1.02	221.27 ± 5.50	76.79 ± 2.86	19.10 ± 1.92	1.37 ± 0.018

### Metal Absorption Capacity Analysis

**Figure 1:**
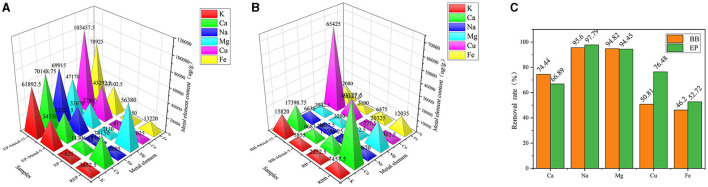
Metal element content of samples (μg/g). **(A)** Metal element content of eucalyptus samples. **(B)** Metal element content of bamboo samples. **(C)** Metal element removal rate of acid wash samples.

The metal absorption capacity of lignocellulose plays an important role in the further catalytic pyrolysis process. Different plant species present different metal absorption ability. In the present study, the contents of metallic elements of raw materials and metal-loaded samples were measured by AAS, and the results are shown in Figure 1. Compared with other investigated metallic elements, Mg and Ca were the two major metallic elements in REP and RBB, which may be attributed to the higher content of Mg/Ca in plant growth environment (Kroukamp et al., 2016). As shown in Figure 1C, the contents of the investigated metallic elements (Ca, K, Na, Mg, Cu, Fe) in eucalyptus and bamboo significantly reduced after acid-washed with 50.72–97.79% decrement in eucalyptus samples, while 46.20–95.60% decrement in bamboo samples. These results reflected that metallic elements in lignocellulose could be efficiently removed by the acid-washed pretreatment, thus reducing the influence of original metallic elements on the subsequent experiments. Moreover, acid-washed could also affect physicochemical properties of lignocellulose by changing the functional groups and complex structure (Dong et al., 2015). However, a certain amount of original metal ions still existed after acid-washed under the investigated conditions. Therefore, the final metallic element content in metal-loaded samples should be considered.

As shown in Figures 1A,B, the content of the investigated metallic elements in the metal-loaded samples was increased significantly with the increment of impregnation concentration. After treated with the highest concentration (15 wt%), the increment of different metallic elements in eucalyptus was 9802.80% (K), 520.44% (Ca), 121491.30% (Na), 1407.03% (Mg), 16654.25% (Cu), and 1162.80% (Fe) in comparison with acid-washed samples (before impregnation), respectively. Different metals had different loaded capacity in the same plant material. Moreover, the content of metallic elements in eucalyptus and bamboo was different even treated under the same conditions. These phenomena may be due to the reason that the transport capacity and the complexing ability of metal ions with functional groups vary among different materials (Krishnani et al., 2008). The lower the ion radius is, the stronger the metal ion transport capacity is. Moreover, the complexing ability of metal ions with function groups in eucalyptus and bamboo varied due to their different natural structure and growth characteristics. In addition, the number of functional groups also plays an important role in the metal absorption capacity of plant materials (Oudenhoven et al., 2015). Metal ion adsorption usually occurs in the presence of highly electronegative atoms, such as O, N, P, S. Lignocellulose is rich in oxygen-containing groups such as carboxyl groups (mainly in hemicellulose), phenolic hydroxyl groups (mainly in lignin), hydroxyl groups, and other electronegative atoms in the existence form of nutrients. In addition, eucalyptus is a hardwood plant whose mainly hemicellulose structure is glucuronoxylan, while bamboo belongs to the grass family and its hemicellulose structure is glucuronoarabinoxylan. Compared with glucuronoarabinoxylan, glucuronoxylan possesses the larger carboxyl amount. Moreover, the hemicellulose content of eucalyptus was higher than that of bamboo. Hence, the metal loading ability of eucalyptus was higher than that of bamboo. Furthermore, we considered that the structure of lignocellulose also played an important role in the metallic element enrichment process during the plant growth. Due to the structural diversity of the three major components of lignocellulose, plants showed enrichment, and storage capability for different metallic elements. However, the absorption of metallic elements was a complex process for lignocellulose, which may be relevant to many factors, such as their natural structure, nutrient content, and chemical composition.

### Catalytic Pyrolysis Behavior

**Figure 2:**
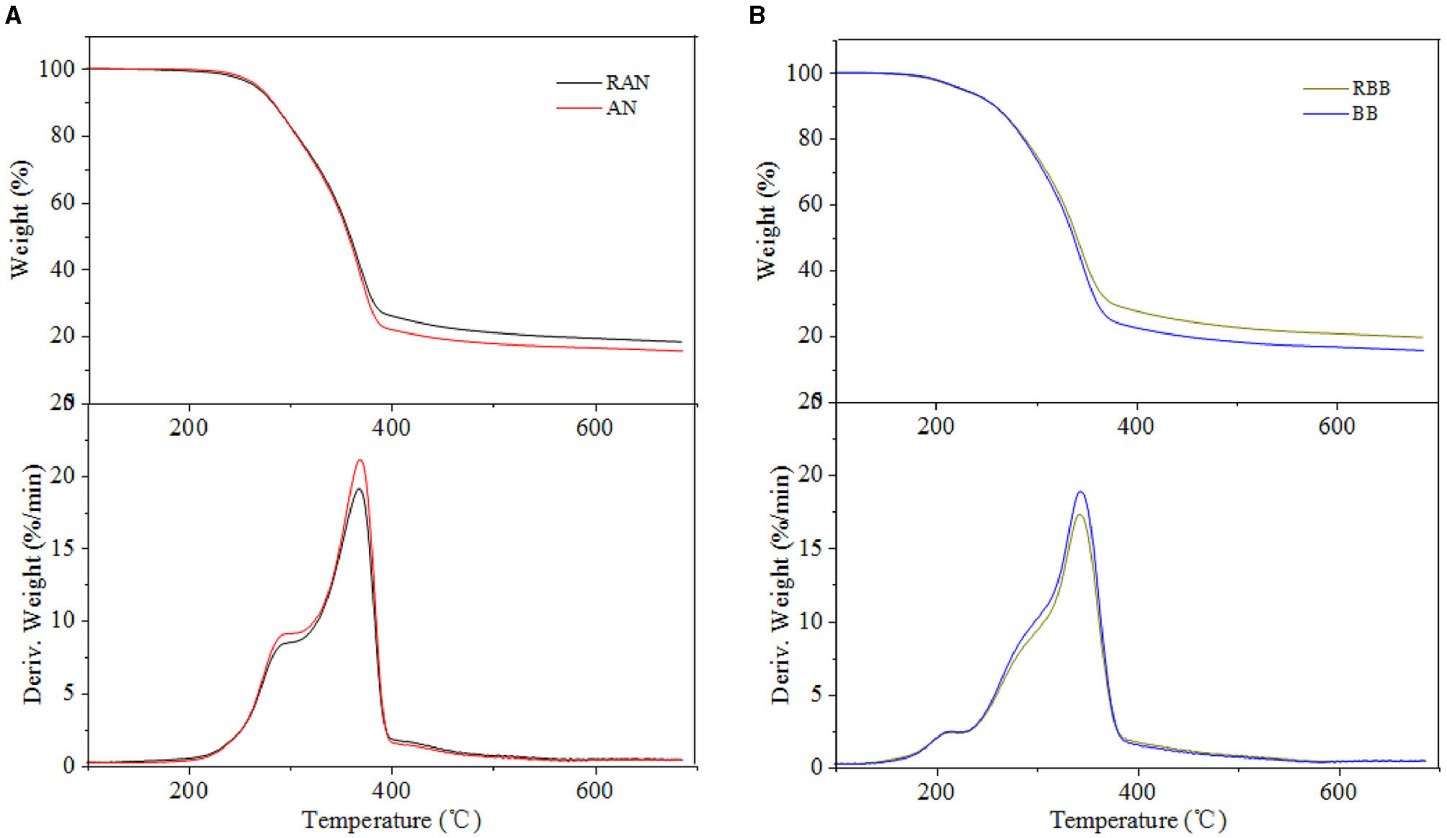
**(A,B)** TG and DTG curves of raw material and acid-washed samples.

**Figure 3:**
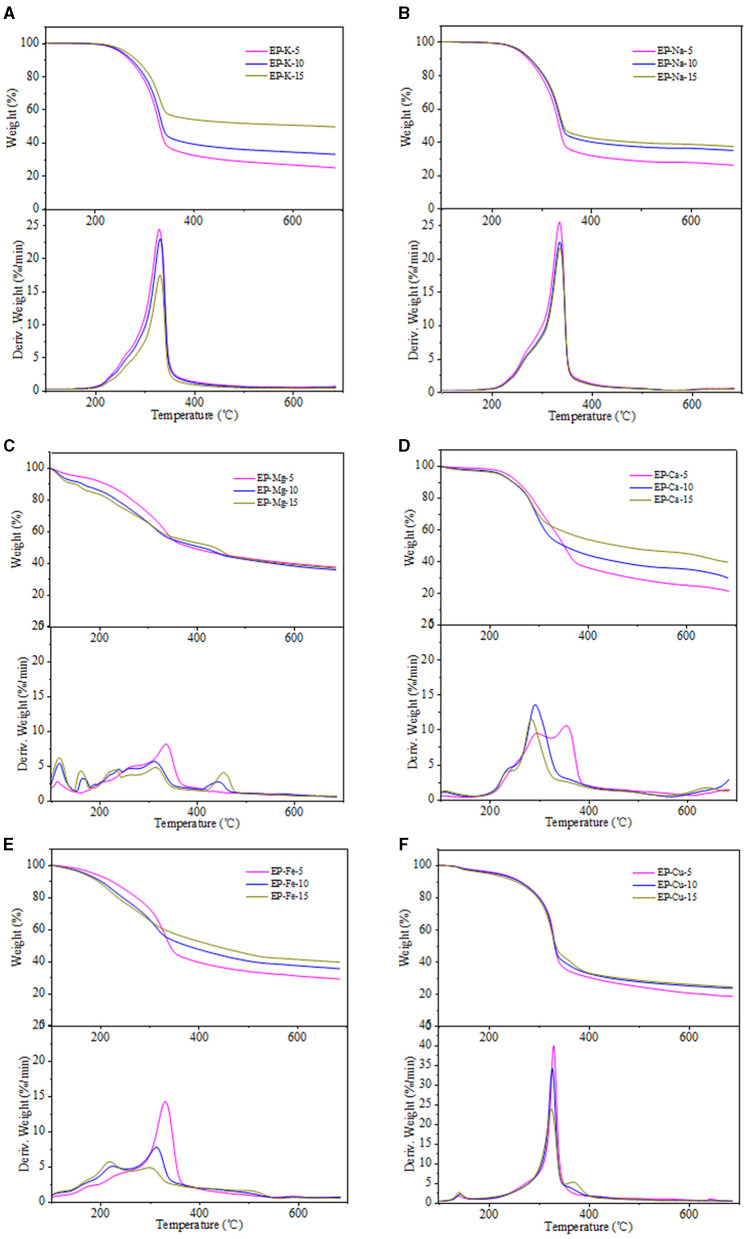
**(A–F)** TG and DTG curves of metal loaded eucalyptus samples.

**Figure 4:**
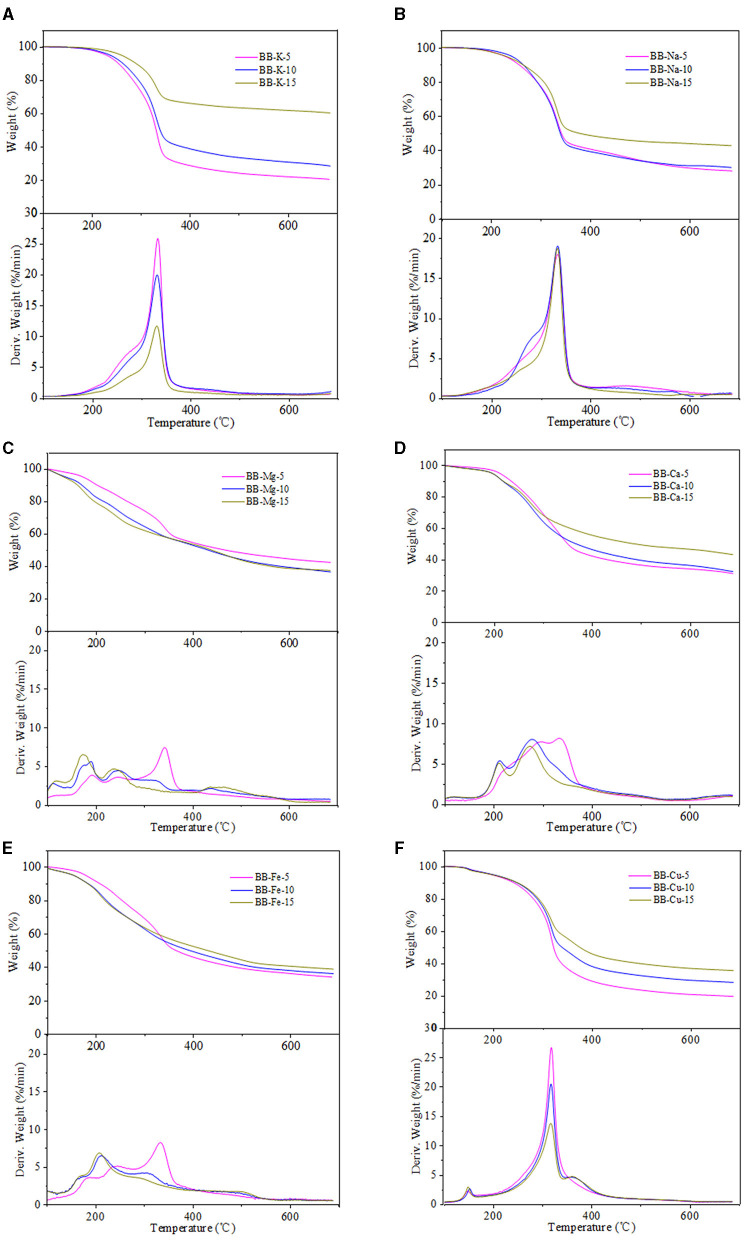
**(A–F)** TG and DTG curves of metal loaded bamboo samples.

The pyrolysis behavior of metal-loaded eucalyptus and bamboo samples was evaluated by TG and DTG, and the results are shown in Figures 2–4. Generally, the thermal decomposition of lignocellulose could be divided into three stages (Li et al., 2016). In the first stage, the absorbed water lost near 100°C. The second stage was the fast pyrolysis stage to generate volatile compounds. The decomposition temperature range of eucalyptus and bamboo in the second stage was 220–398°C and 190–375°C, respectively. The third stage at the temperature up to 400°C corresponded to the formation of biochar. The degradation rates of REP and RBB during the second pyrolysis stage were 78 and 75%, respectively, which was in good agreement with the volatile matter results in Table 1. The weight loss of the investigated biomass was resulted from the decomposition of cellulose, hemicellulose, and partial lignin to produce CO_2_, CO, CH_4_, and other products. According to the literature, the weight loss of hemicellulose occurred at 220–315°C, while the pyrolysis of cellulose focused at a higher temperature range of 315–400°C (Yang et al., 2007). However, lignin pyrolyzes at the wider temperature ranging from 160 to 900°C (Yang et al., 2007). In comparison with REP and RBB, the weight loss of acid-washed materials (EP and BB) in the second stage was larger than that of raw materials (REP and RBB), which may be assigned to the fact that acid-washed could destroy the surface of materials and partly hydrolyze hemicellulose and cellulose (Dong et al., 2015). DTG curves also showed that the temperatures corresponding to the highest mass weight loss peak of eucalyptus-based materials were higher than those of bamboo-based materials even treated at the same conditions, indicating that the fast pyrolysis process of eucalyptus occurred at the higher temperature. This phenomenon may be mainly ascribed to the different contents of hemicellulose and crystalline cellulose between bamboo and eucalyptus. In addition, eucalyptus is a hardwood plant whose main hemicellulose structure is glucuronoxylan, while bamboo belongs to the grass family and contains glucuronoarabinoxylan. The hemicellulose side chain and the degree of branching could affect its thermal stability. The higher branching degree of hemicellulose is, the lower thermal stability could be achieved (Dai et al., 2021). The difference in hemicellulose structure may be one of the reasons for the various thermal stabilities between eucalyptus and bamboo (Werner et al., 2014). In addition, the temperature of the maximum weight loss rate in the DTG curves was reduced with the reduction of cellulose crystallinity, which may be due to the reason that the lower crystallinity of cellulose has less number of hydrogen bonds needed to be broken (Leng et al., 2020).

The pyrolysis residue weights of raw materials (REP and RBB) after 400°C were higher than those of acid-washed samples, which may be ascribed to the removal of inorganic compounds by the acid-washed approach. Moreover, the difference of hemicellulose structure and lignin methoxy groups content between eucalyptus and bamboo could also influence the final char yield. The quality of the residue was negatively correlated with the methoxy group content in lignin. Less methoxy groups led to the higher pyrolysis residue quality. Bamboo lignin includes three types: *p*-hydroxyphenyl lignin, guaiacyl lignin, and sinapyl lignin, while eucalyptus lignin was composed of guaiacyl lignin and sinapyl lignin (Wen et al., 2013). This was likely to cause the methoxy content of bamboo to be lower than that of eucalyptus, resulting in the higher residual carbon content. In addition, the pyrolysis gas product CO_2_ was mainly released from -COOH and C=O in hemicellulose (Yang et al., 2007). Compared with bamboo, eucalyptus hemicellulose contained more -COOH, which could release more CO_2_ and reduce the pyrolysis residue weight. Compared with hemicellulose, lignin had a greater effect on the pyrolysis residue content. Specifically, the higher the lignin content was, the larger the quality of pyrolysis residue could be obtained (Yang et al., 2007).

As shown in Figures 3, 4, the temperatures corresponded to the initial devolatilization and the highest weight loss rate of metal-loaded samples was decreased significantly in comparison with the acid-washed materials, indicating that the existence of investigated metal ions could accelerate the pyrolysis process of EP and BB (Shimada et al., 2008). This phenomenon was in good agreement with the following Ea results. Moreover, different metal ions at the same concentration have different effects on the thermal degradation of lignocellulose. The influence of the investigated metal ions on the reduction of initial devolatilization temperature of eucalyptus-based and bamboo-based materials was in order of Mg-form > Fe-form > Ca-form > Cu-form > K-form > Na-form. In terms of TG curves, the pyrolysis residue weight of eucalyptus and bamboo samples was increased with the increment of metal impregnation concentration, except for Mg-loaded samples. The existence of metal ions may hinder the evaporation of volatiles, thus enhancing the quality of pyrolysis residues (Xiao et al., 2020). Therefore, the generation and release of pyrolysis steam reduced, and the yield of biochar increased. Moreover, this phenomenon may be related to the characteristics of metallic elements. Na and K are alkali metals, Mg and Ca are alkaline earth metals, and Cu and Fe are transition metals. Compared with Na and K, other metal ions presented the better oxidizability, thus promoting the decomposition of lignocellulose. The maximum weight loss rate of 5% metal-loaded samples was larger than that of 10 and 15% metal-loaded materials, which indicated that the lower concentration of the investigated metallic elements was likely to promote the pyrolysis of carbohydrates in lignocellulose. Furthermore, the DTG curves showed that Mg-loaded samples exhibited more shoulders than other materials, which may be ascribed to the strong integration between bounded water and MgCl_2_. Bounded water of MgCl_2_·XH_2_O was difficult to be removed even at boiling point. In addition, Mg undergoes many changes (MgCl·4H_2_O, MgCl_2_·2H_2_O, Mg(OH)Cl, MgCl_2_, MgO) in the process of pyrolysis and produces HCl (Shimada et al., 2008)_._ Fe and Cu also had the ability to generate hydrates, and DTG curves of Fe/Cu-loaded materials were similar to those of Mg-loaded samples. Among all the investigated metallic elements, Mg showed the best catalytic performance for facilitating the pyrolysis process of eucalyptus and bamboo. It should be noted that the existence form of metallic elements changed and can catalyze the pyrolysis of lignocellulose. We speculated that different internal metallic elements of plants will cause significant differences in their subsequent utilization process.

### Fourier-Transform Infrared Analysis

**Figure 5:**
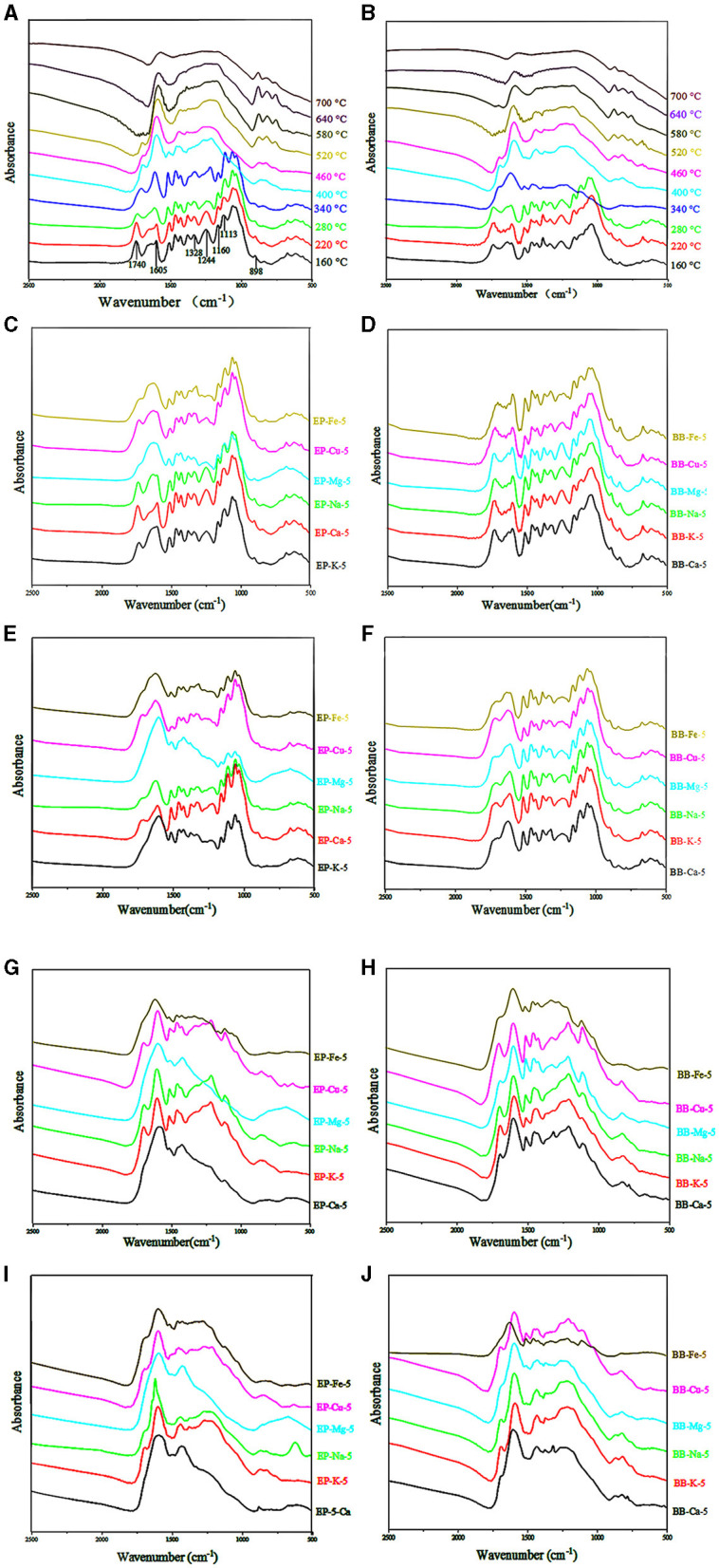
Different samples FT-IR spectrums. **(A)** IR spectral of EP with different pyrolysis temperature. **(B)** IR spectral of BB with different pyrolysis temperature. **(C)** IR spectral of EP-5 which pyrolysis temperature was 220°C. **(D)** IR spectral of BB-5 which pyrolysis temperature was 220°C. **(E)** IR spectral of EP-5 which pyrolysis temperature was 280°C. **(F)** IR spectral of BB-5 which pyrolysis temperature was 280°C. **(G)** IR spectral of EP-5 which pyrolysis temperature was 340°C. **(H)** IR spectral of BB-5 which pyrolysis temperature was 340°C. **(I)** IR spectral of EP-5 which pyrolysis temperature was 400°C. **(J)** IR spectral of BB-5 which pyrolysis temperature was 400°C.

Chemical structural changes of eucalyptus and bamboo samples during the pyrolysis temperature ranged from 160 to 700°C were analyzed by FT-IR, and the results are shown in Figure 5. Signals of typical functional groups in the pyrolyzed samples are listed in Table 2. The peak at 898 cm^−1^ is assigned to the C-H vibration of β-glucosidic linkage between sugar units disappeared when the temperature was up to 400°C (eucalyptus) and 340°C (bamboo), which suggested that the destruction of sugar unit linkage may take place at high temperature. Strong band at 1,740 cm^−1^ that corresponded to the C=O stretching vibration in the acetyl groups of hemicellulose was weakened or even missed when the temperature was up to 280°C. Absorption at 1,160 cm^−1^ originated from the ester bond of glucuronoarabinoxylan disappeared at the relatively high temperature (> 400°C). These results indicated that the thermal stability of ester bond of glucuronoarabinoxylan was higher than that of acetyl groups. Therefore, acetyl groups may be decomposed prior to ester bond of glucuronoarabinoxylan during the pyrolysis process. Moreover, the intensity of the vibration at 898 cm^−1^, 1,160 cm^−1^, and 1,740 cm^−1^ related to the carbohydrates was decreased sharply when the pyrolysis temperature was increased from 220 to 400°C, which indicated that 220–400°C was the major degradation interval for carbohydrates during the pyrolysis process. These results were in good agreement with the TG results.

**Table 2:** The main functional groups of the solid pyrolysis residues.

**Wavenumber (cm^**−1**^)**	**Description of functional groups**	**Compounds**	**References**
898	C-H vibration	β-Glucosidic linkage between sugar units	Peng et al., 2010
1,113	C-H deformation	Lignin	Villaverde et al., 2009
1,160	C-O stretching	Ester bond of arabinoxylan	Bilba and Ouensanga, 1996
1,244	C-O	Lignin	Siengchum et al., 2013
1,328	C-O bending	Lignin	Bilba and Ouensanga, 1996
1,605	Aromatic ring vibration and C=O stretching	Lignin	Xu et al., 2006
1,740	C=O stretching	Hemicellulose	Siengchum et al., 2013

Peak at 1,113 cm^−1^ corresponded to the C-H deformation of lignin. Signals at 1,244 cm^−1^ and 1,328 cm^−1^ were assigned to the C-O linkage of lignin. There was no significant change for the bands at 1,328 cm^−1^ of eucalyptus lignin in the pyrolysis temperature of 160–400°C, while the absorption band at 1,244 cm^−1^ disappeared at 400°C. The intensity of the lignin signals was reduced with the increment of pyrolysis temperature from 400 to 700°C. Interestingly, the strength of the peak at 1,605 cm^−1^ enhanced first and then decreased, which may be attributed to the change in lignin content during pyrolysis. Since the thermal stability of lignin was higher than that of carbohydrates, the relative content of lignin was initially increased and then decreased during the pyrolysis process.

The intensity of absorption bands of different metal-loaded eucalyptus materials remained stable within 220°C (Figures 5C–J). However, when the pyrolysis temperature increased from 280 to 340°C, the signals related to carbohydrates and lignin of Mg-, Ca-, and Fe-loaded eucalyptus weakened gradually, and the carbohydrate-related peaks decreased significantly. Compared with K, Na, and Cu, the intensity of the absorption bands of Mg-, Fe-, and Ca-loaded samples decreased more obviously when the pyrolysis temperature was up to 340°C. This phenomenon was consistent with the phenomenon that the catalytic ability of Mg, Fe, and Ca for the pyrolysis of eucalyptus and bamboo was better than that of K, Na, and Cu.

### Kinetic Analysis

**Figure 6:**
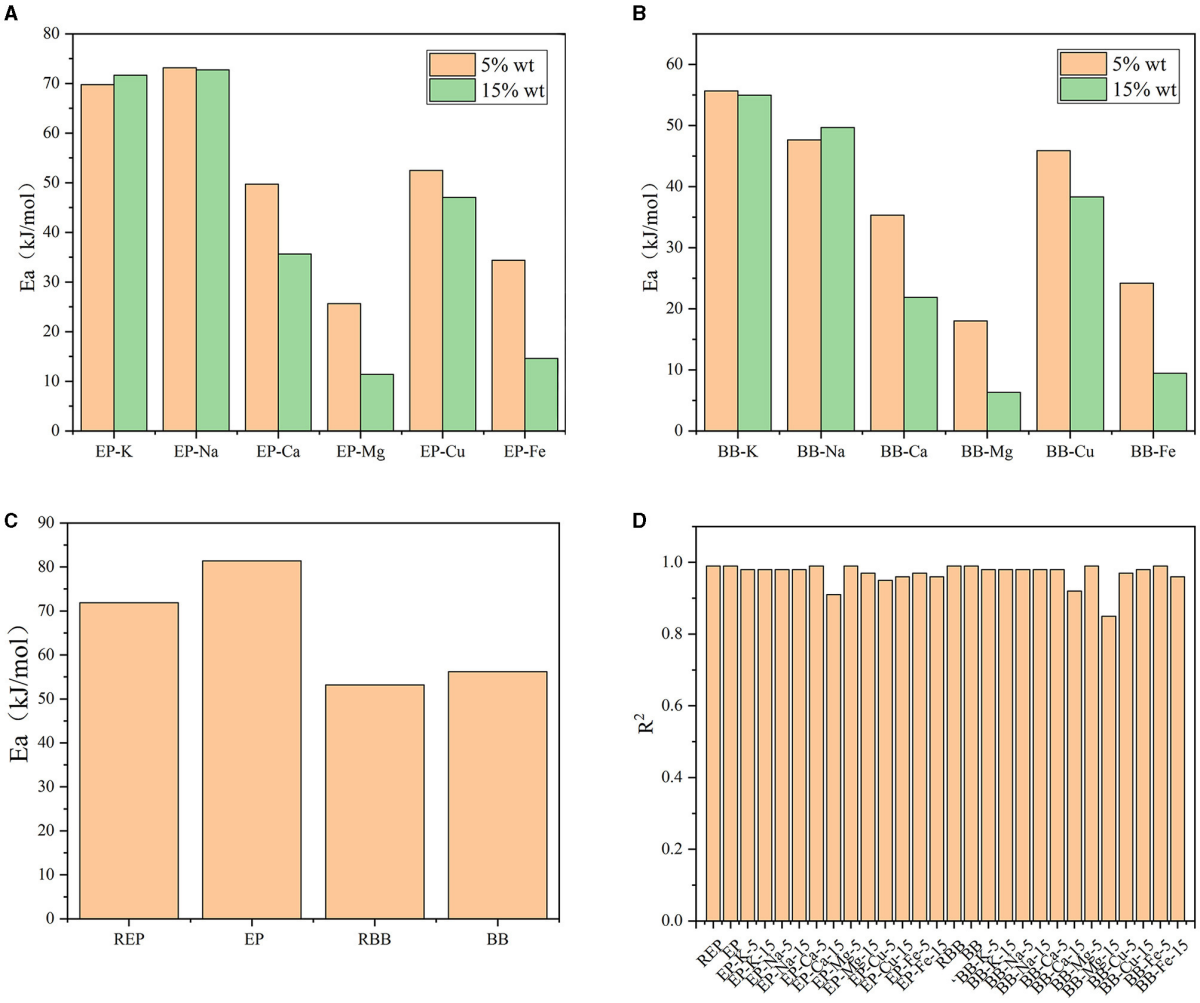
**(A)** Ea of AM-loaded eucalyptus samples during pyrolysis process. **(B)** Ea of AM-loaded bamboo samples during pyrolysis process. **(C)** Ea of REP, EP, RBB, BB during pyrolysis process. **(D)** *R*^2^ of the Kinetic models.

In order to achieve a better understanding of the carbohydrates decomposition stage at the temperature range of 220–400°C, apparent activation energy (Ea) of the reactions was calculated according to Equations 7 and 8, and the results are shown in Figure 6. Results showed that the kinetic models had a good fit with *R*^2^ over 0.85. Compared with REP and RBB, Ea of EP and BB increased after acid-washed, which may be contributed to the fact that the acid-washed treatment could lower the intrinsic metallic elements content of raw materials. In comparison with bamboo, more energy was required for the pyrolysis of eucalyptus-based materials when treated under the same conditions, which may be due to the tighter structure of eucalyptus. Hence, the pyrolysis of second stage of eucalyptus-based materials occurred at the higher temperature than that of bamboo-based materials, which was consistent with the TG analysis. Among the investigated metallic elements, K and Na had a slight promoting effect on the rapid pyrolysis stage (mainly carbohydrates decomposition) of eucalyptus and bamboo, which was in good agreement with the literature (Cai et al., 2017). However, the presence of Mg, Fe, Cu, and Ca could effectively promote the pyrolysis of carbohydrates in eucalyptus and bamboo, and the Ea value decreased as the concentration increased. Mg and Fe showed the best catalytic performance for the pyrolysis of eucalyptus and bamboo, which may be due to the existence form of Mg and Fe in plants. Mg/Fe may complex with functional groups in lignocellulose, thus promoting the pyrolysis process *in situ* directly. Therefore, we presumed that some kind of plant absorbed metallic elements can be used fully to enhance its pyrolysis efficiency.

## Conclusions

In summary, the dynamic changes of thermal behavior during the pyrolysis of eucalyptus and bamboo were investigated under the catalyzation of six kinds of plant-rich metallic elements. Results showed that eucalyptus presented the better metal absorption capacity and thermal stability in comparison with bamboo. Compared with acid-washed materials (EP and BB), the temperatures corresponded to the initial devolatilization and the highest weight loss rate of metal-loaded samples decreased in the order of Mg > Fe > Ca > Cu > K > Na. The existence form of metallic elements changed and can catalyze the pyrolysis of lignocellulose. FT-IR results showed that the thermal stability of ester bond of glucuronoarabinoxylan was higher than that of acetyl groups, leading to the easier decomposition of acetyl groups in hemicellulose during pyrolysis. Kinetic analysis indicated that Mg and Fe could greatly accelerate the pyrolysis process of eucalyptus and bamboo with a reduction of Ea. Compared with bamboo, more energy was required for the pyrolysis of eucalyptus-based materials when treated under the same conditions. Further study was in progress to study the influence of plant cultivation conditions on its pyrolysis behavior.

## Data Availability Statement

The original contributions presented in the study are included in the article/supplementary material, further inquiries can be directed to the corresponding author/s.

## Author Contributions

JH involved in investigation, data curation, and writing the original draft. BZ involved in formal analysis and involved in using software. ZH, PO, and YL involved in formal analysis. AW involved in resources, methodology, supervision, writing the review and editing, and funding acquisition. HL involved in conceptualization, methodology, writing the review and editing, supervision, and funding acquisition. All authors contributed to the article and approved the submitted version.

## Funding

This work was supported by grants from the Program for National Natural Science Foundation of China (No. 31700506) and the Natural Science Foundation of Guangdong Province, China (2017A030310550 and 2020A1515011009). This work was successful by the support of program for innovative research team of Huizhou University.

## Conflict of Interest

The authors declare that the research was conducted in the absence of any commercial or financial relationships that could be construed as a potential conflict of interest. The reviewer HP declared a shared affiliation, though no other collaboration, with several of the authors JH, BZ, ZH, PO, AW, and HL to the handling Editor.

## Publisher's Note

All claims expressed in this article are solely those of the authors and do not necessarily represent those of their affiliated organizations, or those of the publisher, the editors and the reviewers. Any product that may be evaluated in this article, or claim that may be made by its manufacturer, is not guaranteed or endorsed by the publisher.
